# Biosynthetic Plasticity Enables Production of Fluorinated Aurachins

**DOI:** 10.1002/cbic.202000166

**Published:** 2020-05-05

**Authors:** Angela Sester, Katrin Stüer‐Patowsky, Wolf Hiller, Florian Kloss, Stephan Lütz, Markus Nett

**Affiliations:** ^1^ Department of Biochemical and Chemical Engineering Laboratory of Technical Biology TU Dortmund Emil-Figge-Strasse 66 44227 Dortmund Germany; ^2^ Department of Chemistry and Chemical Biology NMR Laboratory TU Dortmund Otto-Hahn-Strasse 4a 44227 Dortmund Germany; ^3^ Transfer Group Antiinfectives Leibniz Institute for Natural Product Research and Infection Biology Beutenbergstrasse 11a 07745 Jena Germany; ^4^ Department of Biochemical and Chemical Engineering Chair for Bioprocess Engineering TU Dortmund Emil-Figge-Strase 66 44227 Dortmund Germany

**Keywords:** aurachin, biosynthesis, biotransformation, myxobacteria, *Stigmatella*

## Abstract

Enzyme promiscuity has important implications in the field of biocatalysis. In some cases, structural analogues of simple metabolic building blocks can be processed through entire pathways to give natural product derivatives that are not readily accessible by chemical means. In this study, we explored the plasticity of the aurachin biosynthesis pathway with regard to using fluoro‐ and chloroanthranilic acids, which are not abundant in the bacterial producers of these quinolone antibiotics. The incorporation rates of the tested precursor molecules disclosed a regiopreference for halogen substitution as well as steric limitations of enzymatic substrate tolerance. Three previously undescribed fluorinated aurachin derivatives were produced in preparative amounts by fermentation and structurally characterized. Furthermore, their antibacterial activities were evaluated in comparison to their natural congener aurachin D.

## Graphical abstract

**Room for a halogen**: The plasticity of the aurachin biosynthetic pathway in the myxobacterium *S. erecta* was explored for the use of fluoro‐ and chloroanthranilic acids. Incorporation of the unnatural precursors was quantified. Three fluorinated aurachin analogues were produced in sufficient quantity to enable their antibacterial activities to be assessed.

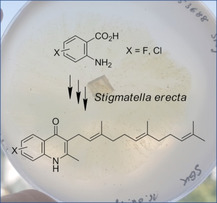

Nature is a rich source of bioactive compounds, which have been optimized during evolution regarding their affinity to cellular targets.^1^ In drug development, such “privileged” scaffolds are often derivatized in order to identify more active, less toxic or metabolically more stable compounds. The necessary structural modifications can be implemented by semi‐ and total synthesis or, alternatively, by biotechnological means. A proven method is to introduce analogues of natural starter units or intermediates into a biosynthetic pathway.^2^, ^3^, ^4^, ^5^ This is usually achieved by feeding the native producer strain or an engineered block mutant with an unnatural surrogate, which can become incorporated into the natural product provided the corresponding biosynthesis enzymes exhibit low substrate specificity.^6^, ^7^, ^8^, ^9^, ^10^

The aurachins are a large family of prenylated quinolone antibiotics, which were first discovered in cultures of the myxobacterium *Stigmatella aurantiaca* and later also found in other *Stigmatella* spp. as well as members of the actinomycete genera *Rhodococcus* and *Streptomyces*.^[11] ^Their structural relatedness to the known respiratory chain inhibitor 2‐heptyl‐4‐hydroxyquinoline‐*N*‐oxide (HQNO) suggested early on that the aurachins might interfere with electron transport processes.11a This assumption was confirmed in subsequent investigations and it was further demonstrated that the aurachins do not only affect complexes I and III of the respiratory chain, but also photosystem II and the cytochrome *b_6_f* complex.^12^ Despite their potent bioactivities,^11^, ^13^ previous structure‐activity relationship (SAR) studies mainly focused on the variation of the farnesyl side chain in aurachin D (**1**) and C (**2**).11c, 11d, 11e, 14, 15, 16 In contrast, the generation of aurachins with different substituents at C‐5 to C‐8 has been neglected, which is quite surprising in light of the obvious structural similarity to synthetic quinolone antibiotics, such as ciprofloxacin.

To explore this yet underexplored chemical space, we considered precursor‐directed biosynthesis as a viable option to introduce functional groups at these positions. Isotopic labelling studies^17^ as well as biochemical experiments^18^ had previously shown that the aurachins originate from anthranilic acid, which is condensed with two malonyl units upon enzymatic activation (Scheme 1). Therefore, substituted analogues of anthranilic acid appeared to be promising delivery vehicles for the modification of the aurachin core. Initially, we decided to probe the incorporation of fluoro‐ and chloroanthranilic acids, as regiospecific introduction of halogen moieties is challenging by chemical synthesis.^19^ Moreover, the introduction of halogens, particularly fluorine, is often considered during fine‐tuning of lead properties in medicinal chemistry.^20^ Many structurally complex natural products could be endowed with fluorine substituents by chemo‐biosynthetic strategies. Examples include vancomycin‐type antibiotics, such as balhimycin,21a the antibacterial wailupemycins,21b the proteasome inhibitor salinosporamide,21c the antimitotic ansamitocin P‐3,21d or the immunosuppressant rapamycin.21e From a methodological perspective, the feeding of halogenated precursors is also appealing. While chlorinated compounds show characteristic isotope patterns that are easily recognized in mass spectrometry, the incorporation of fluorinated precursor molecules can be followed by ^19^F NMR spectroscopy.

**Scheme 1:**
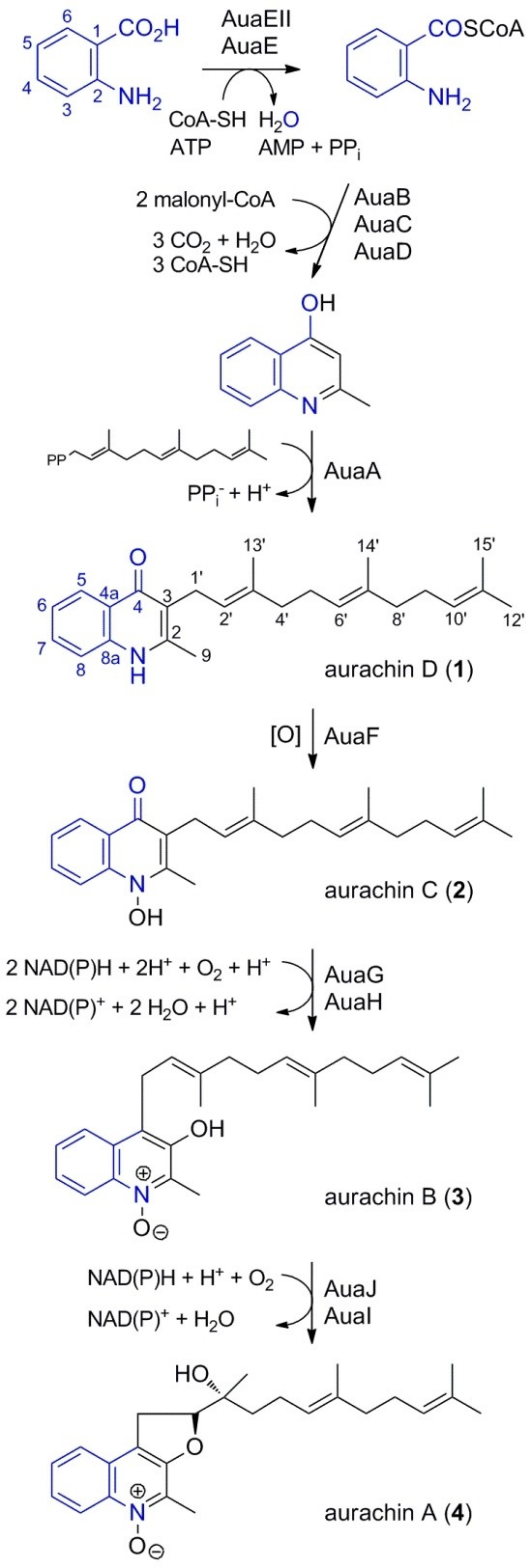
Biosynthetic pathway to the four major aurachins.^16^ The anthranilic acid building block is highlighted in blue.

The feeding studies were conducted in shaken Erlenmeyer flasks with a *Stigmatella erecta* strain, which had been confirmed as an authentic aurachin producer.11b For the efficient recovery of the secreted antibiotics, adsorber resins were added to the production medium prior to inoculation of the cultures. During the incubation period we noticed attenuated growth of *S. erecta* in the presence of halogenated anthranilic acids, irrespective of the fluorine or chlorine substitution pattern. This may hint at interferences with tryptophan metabolism, as recently reported for mycobacteria.^22^ Subsequent to separation of the adsorber resin from the culture broth, elution with acetone and methanol yielded crude extracts suitable for liquid chromatography‐coupled mass spectrometry (LC/MS)‐assisted relative quantification of aurachin derivatives. Previous studies had already indicated that the biosynthesis of individual aurachins is linked to the growth phase of the producing bacterium.11a *S. erecta*, like many other myxobacteria, does not grow homogeneously in liquid media, but is known to form cellular aggregates.^23^ As this feature makes it very difficult to precisely determine the growth stage of *S. erecta*, the amounts of biosynthesized aurachins were normalized to the production of myxothiazol A. This known secondary metabolite from *S. erecta* 11b does not recruit anthranilic acid for its biosynthesis and its production profile is similar to the aurachins.^24^, ^25^ The results of this quantification are illustrated in Figure 1.

**Figure 1:**
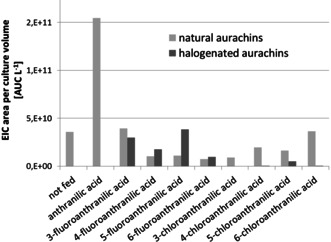
Normalized quantification of aurachins in *S. erecta* cultures after feeding with halogenated anthranilic acids. EIC=extracted ion chromatogram.

In accordance with previous studies, the addition of anthranilic acid was found to have a positive effect on aurachin production.11a The total amount of aurachins increased by a factor of four when compared to unsupplemented cultures, which suggests that the availability of the natural aromatic precursor is a limiting factor in the biosynthesis. In contrast, the cultivation in the presence of halogenated anthranilic acids had a lower impact or even decreased the production level. Moreover, significant differences in the utilization of the unnatural precursors became obvious. While all fluoroanthranilic acids could be converted into novel aurachins, albeit to different extent, the biosynthesis enzymes were obviously much less tolerant with regard to chlorinated starter units. Except for the incorporation of 5‐chloroanthranilic acid, only trace amounts of chloroaurachins were detected by LC/MS. Among the fluorinated precursor analogues, 5‐fluoroanthranilic acid showed the highest conversion, followed by the aromatic acids bearing the halogen substituent in positions 3 and 4, respectively. In comparison, the 6‐fluoroanthranilic acid‐derived aurachins were produced in much lower quantities. These results indicate that the introduction of halogen atoms in aurachin biosynthesis depends both on the nature of the halogen (F>Cl) and on the substitution pattern of the precursor molecule (pos. 5>3>4>6). In none of the feeding settings, the anticipated acyl‐CoA derivatives of halogenated anthranilic acids were found, whereas correspondingly substituted 4‐hydroxy‐2‐methylquinolines were detected at trace levels in cultures supplemented with fluoro‐ as well as 4‐ and 5‐chloroanthranilic acids. Since no accumulation of any intermediate was observed, we hypothesize that substrate discrimination already occurs in the early steps of aurachin biosynthesis.

In order to enable full NMR‐based structural characterization, feeding studies were repeated on multilitre scale to produce sufficient quantities of some aurachin analogues. Owing to the very low production titres that were achieved (Figure S1, Table S1) and a laborious chromatographic separation process (Figure 2), only the most abundant derivatives could be isolated in appreciable amounts. In case of novel fluoroaurachins the purification procedure was significantly facilitated by ^19^F NMR guided fractionation (Figure S2). The recovery of 6‐chloroaurachin D remained unsuccessful despite repeated attempts.

**Figure 2:**
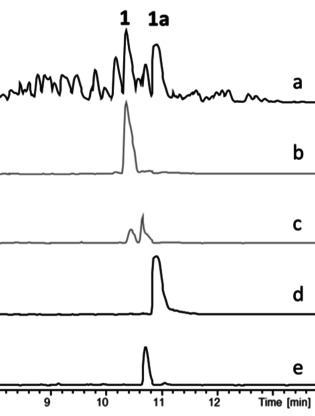
LC/MS analysis of an extract from a *S. erecta* culture fed with 3‐fluoroanthranilic acid. a) Base peak chromatogram (a) and extracted ion chromatograms for **1** (b), **2** and **3** (c), fluorinated **1** (d), as well as fluorinated **2** and/or **3** (e).

Compound **1 a** (0.5 mg) originated from a 10‐L culture of *S. erecta* grown in the presence of 3‐fluoroanthranilic acid. The [*M*+H]^+^ ion peak of **1 a** possesses a *m/z* value of 382.2546, corresponding to a molecular formula of C_25_H_32_NOF and 10 double bond equivalents. UV maxima at 241, 321 and 333 nm as well as NMR data (Figures S3–S7) of the purified compound are almost consistent with those of **1**,11a, 17 which suggested a closely related molecular architecture. As expected, the ^1^H NMR spectrum of **1 a** features only three aromatic signals with a distinctive coupling pattern characteristic for a 3‐fluoroanthranilate‐derived moiety. COSY and HMBC experiments confirmed the presence of a farnesyl chain and a methyl group, which are connected to the assumed quinolone core of **1 a** at C‐3 and C‐2, respectively. Thus, **1 a** was identified as 8‐fluoroaurachin D.

The extract from the 4‐fluoroanthranilic acid culture yielded 0.3 mg of compound **1 b** after multi‐step HPLC. The high‐resolution ESI‐MS spectrum confirmed the same molecular formula as **1 a** as well as similar UV maxima at 249, 321 and 331 nm (Figure S8). A comparison of ^1^H NMR spectra corroborated that **1 a** and **1 b** are indeed structurally similar, but not identical. The differences pertained to the aromatic region. Following an analysis of their multiplicities and coupling constants, the aromatic proton signals of **1 b** could be assigned to a spin system for a 1,2,4‐trisubstituted benzene ring. Further interpretation of 1D and 2D NMR data (Figures S9–S12) then led to the conclusion that **1 b** represents 7‐fluoroaurachin D.

Compound **1 c** was purified from a culture that had been supplied with 5‐fluoroanthranilic acid, yielding 0.3 mg of material. Again, high‐resolution ESI‐MS and UV data pointed to an aurachin D derivative bearing a single fluorine substituent (Figure S13). The substitution pattern with the halogen at C‐6 position of the quinolone ring was deduced from the heteronuclear coupling observed in the ^1^H NMR spectrum (Figures S14–S17).

Having these novel unnatural derivatives at hand, structure‐activity relationship studies appeared feasible. The isolated compounds were tested in an agar diffusion assay to assess their antibiotic activities against a panel of four bacterial test strains. This analysis revealed that the generated derivatives **1 a**–**c** are equipotent to **1** (Table 1). Thus, fluorine introduction does not significantly alter the observed bioactivity profile. In comparison to the reference ciprofloxacin, however, the antibacterial activities of **1** and its analogues turned out to be comparatively weak. Furthermore, we noted that the aurachins only show partial inhibition against the test bacteria. This observation might indicate spontaneous resistance.

**Table 1:** Aurachins isolated in this study and their antibacterial activities in the agar diffusion assay.

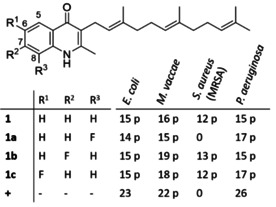

The given values represent the diameters of the respective inhibition zone in mm. The aurachins were tested at a concentration of 0.5 mg mL^−1^, whereas ciprofloxacin was tested at a concentration of 0.005 mg mL^−1^. Abbreviations: **+**, ciprofloxacin; p, partial inhibition (= few colonies within the inhibition zone).

This study shows for the first time that the aurachin pathway exhibits sufficient plasticity to enable precursor‐directed biosynthesis. The biosynthetic enzymes generally accepted fluorine substitution at the anthranilic acid precursor, but were less permissive with regard to the more bulky chlorine atom. Furthermore, we demonstrated that the C‐5 position of the anthranilic acid precursor is particularly suited for the introduction of functional groups. We therefore assume that our strategy may be of more general use for more in depth systematic SAR investigations. Although three fluoroaurachins were successfully generated and structurally and biologically characterized in this study, it is evident from their titres that the production conditions were not favourable. This is in part due to the replacement of the originally described production medium,11b which is no longer commercially available. In other part it is due to the biosynthetic dispersion into several products including a number of minor aurachins in addition to **1**–**4** and their halogenated variants.^17^

To improve the aurachin yields different approaches are conceivable. The genetic inactivation of late‐stage pathway enzymes in *S. erecta* could lead to an accumulation of early biosynthetic intermediates, thereby reducing the chemical complexity of the respective extracts. Heterologous expression of the entire or partial aurachin gene cluster provides the means to bypass natural regulation and, hence, to increase the yield. To overcome growth inhibition by anthranilic acid analogues, a strain could be chosen that is more resistant to these building blocks. Alternatively, the enzymatic conversion of anthranilic acid into tryptophan could be disrupted. In this way, unnatural anthranilic acids could not be recruited for tryptophan generation and, therefore, not interfere with primary metabolism.^26^ The inactivation of *S. erecta* anthranilate synthase could further eliminate the competition with the natural precursor and hence enable mutasynthesis. The feasibility of such an approach is, however, hardly predictable. Previous studies in the field targeted only non‐essential pathways to dedicated building blocks of secondary metabolism, such as 3‐hydroxy‐, 3‐hydroxy‐4‐methyl‐, or 3‐amino‐5‐hydroxy‐anthranilic acid.^27^

## Experimental Section

**Culture conditions and incorporation studies**: *S. erecta* Pd e32 (DSM 53688) was grown in modified Zein medium [1 % corn starch (Maizena, Unilever), 0.1 % MgSO_4_×7H_2_O, 0.1 % Bacto Peptone, 0.5 mg L^−1^ vitamin B_12_]. To facilitate the recovery of secreted aurachins, adsorber resin XAD7HP (2 % *w*/*v*) was added prior to sterilization. The medium was inoculated with seed culture (10 % *v*/*v* inoculum) and the fermentation was conducted in an incubator shaker at 30 °C and 130 rpm for 7 days. Anthranilic acid or its halogenated derivatives were supplemented as filter‐sterilized aqueous solutions (33 mg L^−1^) after inoculation of the medium. For extraction the adsorber resin was separated from the culture broth by filtration, washed with water and retained compounds eluted three times with acetone and methanol. The extract was dried and used for relative quantification or compound purification.

**Quantitative analysis**: For relative quantification experiments, the dried extracts of 1.5 L cultures were resuspended in methanol, filtered and supplied to LC‐ESI‐MS (Agilent 1260 Infinity HPLC system combined with a Bruker Daltonics Compact quadrupole‐time of flight mass spectrometer) in positive mode. HPLC flow rate was 0.4 mL min^−1^ on a Nucleodur RP 18 ec column (100×2 mm, 2.7 μm; Macherey‐Nagel) and a gradient from 5 to 98 % acetonitrile in water supplemented with 0.1 % formic acid over 10 min, followed by 5 min at 98 % acetonitrile. All analyses were carried out at a capillary voltage of 4.5 kV, desolvation gas (N_2_) temperature of 220 °C, with a dry gas (N_2_) flow rate of 12 L min^−1^.

**Compound purification**: Dried raw extracts were resuspended in 60 % aqueous methanol (100 mL) and extracted three times with dichloromethane (60 mL). Aurachins were exclusively present in the dichloromethane fraction. The purification continued on a Shimadzu HPLC system (LC‐20AD) equipped with a diode array detector (SPD‐M20A). The initial separation was carried out on a VarioPrep C18 Gravity column (125×0 mm, 5 μm; Macherey‐Nagel) by two consecutive isocratic elutions with pure methanol and 90 % aqueous methanol, respectively. The aqueous eluent was supplemented with 0.1 % (*v*/*v*) trifluoroacetic acid and the flow rates were set to 4 mL min^−1^. For **1 c**, an additional purification on a Nucleodur gravity column (250×10 mm, 3 μm; Macherey‐Nagel) was performed (60 % to 100 % acetonitrile in water supplemented with 0.1 % (*v*/*v*) trifluoroacetic acid within 10 min, followed by 100 % acetonitrile for additional 10 min), at a flow rate of 2 mL min^−1^. NMR spectra were acquired at 300 K on a Bruker AV 700 Avance III HD (CryoProbe) or on a Bruker AV 600 Avance III HD (CryoProbe). Compounds were dissolved in methanol‐*d_4_*, which also served as internal standard to calibrate spectra to *δ*_H_=3.31 ppm and *δ*_C_=49.0 ppm. ^19^F NMR spectra were referenced to residual trifluoroacetic acid at *δ*_F_=−76.5 ppm.

**8‐Fluoroaurachin D (1 a)**: ^1^H NMR (600 MHz, [D_4_]MeOD, 300 K): *δ*=8.02 (dd, *J*_H,H_=7.9, 1.3 Hz, 1H; CH‐5), 7.43 (ddd, *J*_H,H_=7.9, 1.3, *J*_H,F_=11.1 Hz, 1H, CH‐7), 7.30 (dt, *J*_H,H_=7.9, *J*_H,F_=4.9 Hz, 1H, CH‐6), 5.09 (dt, *J*_H,H_=6.9, 1.2 Hz, 1H, CH‐2′), 5.04 (dt, *J*_H,H_=6.9, 1.4 Hz, 1H, CH‐6′), 4.98 (ddt, *J*_H,H_=7.0, 1.5, 1.4 Hz, 1H, CH‐10′), 3.40 (d, *J*_H,H_=6.9 Hz, 2H, CH_2_‐1′), 2.51 (s, 3H, CH_3_‐9), 2.10 (m, 2H, CH_2_‐5′), 2.03 (m, 2H, CH_2_‐4′), 1.92 (m, 2H, CH_2_‐9′), 1.87 (m, 2H, CH_2_‐8′), 1.81 (d, *J*_H,H_=1.2 Hz, 3H, CH_3_‐13′), 1.60 (d, *J*_H,H_=1.4 Hz, 3H, CH_3_‐12′), 1.55 (d, *J*_H,H_=1.4 Hz, 3H, CH_3_‐14′), 1.49 ppm (d, *J*_H,H_=1.5 Hz, 3H, CH_3_‐15′); ^13^C NMR (150 MHz, [D_4_]MeOD, 300 K): *δ*=177.8 (C‐4), 153.3 (d, *J*_C,F_=247.1 Hz, C‐8), 150.4 (C‐2), 136.1 (C‐3′), 135.9 (C‐7′), 132.0 (C‐11′), 130.1 (C‐8a), 127.1 (C‐4a), 125.3 (C‐6′), 125.3 (C‐10′), 123.9 (d, *J*_C,F_=6.9 Hz; C‐6), 123.5 (C‐2′), 121.7 (C‐3), 122.0 (d, *J*_C,F_=4.0 Hz; C‐5), 116.7 (d, *J*_C,F_=16.9 Hz; C‐7), 40.8 (C‐8′), 40.7 (C‐4′), 27.8 (C‐9′), 27.2 (C‐5′), 25.8 (C‐12′), 24.8 (C‐1′), 18.0 (C‐9), 17.6 (C‐15′), 16.3 (C‐13′), 16.2 ppm (C‐14′); ^19^F NMR (600 MHz, [D_4_]MeOD, 300 K): *δ*=133.5 ppm (dd, *J*_F,H_=11.3, 4.8 Hz, 1F, CF‐8); HRMS (ESI): *m/z* calcd for C_25_H_32_FNO: 382.2541 &bk:[*M*+H]^+^; found: 382.2546.

**7‐Fluoroaurachin D (1 b)**: ^1^H NMR (700 MHz, [D_4_]MeOD, 300 K): *δ*=8.25 (dd, *J*_H,H_=8.7, *J*_H,F_=6.2 Hz, 1H; CH‐5), 7.16 (dd, *J*_H,H_=2.4, *J*_H,F_=9.7 Hz, 1H, CH‐8), 7.11 (dt, *J*_H,H_=8.7, 2.4, *J*_H,F_=8.7 Hz, 1H, CH‐6), 5.09 (dt, *J*_H,H_=7.0, 1.4 Hz, 1H, CH‐2′), 5.05 (dt, *J*_H,H_=7.0, 1.4 Hz, 1H, CH‐6′), 4.99 (ddt, *J*_H,H_=7.1, 1.5, 1.4 Hz, 1H, CH‐10′), 3.38 (d, *J*_H,H_=7.0 Hz, 2H, CH_2_‐1′), 2.45 (s, 3H, CH_3_‐9), 2.10 (m, 2H, CH_2_‐5′), 2.02 (m, 2H, CH_2_‐4′), 1.94 (m, 2H, CH_2_‐9′), 1.87 (m, 2H, CH_2_‐8′), 1.80 (d, *J*_H,H_=1.4 Hz, 3H, CH_3_‐13′), 1.61 (d, *J*_H,H_=1.4 Hz, 3H, CH_3_‐12′), 1.55 (d, *J*_H,H_=1.4 Hz, 3H, CH_3_‐14′), 1.51 ppm (d, *J*_H,H_=1.5 Hz, 3H, CH_3_‐15′); ^13^C NMR (175 MHz, [D_4_]MeOD, 300 K): *δ*=177.9 (C‐4), 165.8 (d, *J*_C,F_=240.0 Hz, C‐7), 150.0 (C‐2), 141.4 (C‐8a), 135.9 (C‐7′), 135.7 (C‐3′), 132.0 (C‐11′), 129.5 (C‐5),125.4 (C‐10′), 125.2 (C‐6′), 123.9 (C‐2′), 121.9 (C‐4a), 120.8 (C‐3), 113.3 (C‐6), 103.4 (C‐8), 40.8 (C‐4′), 40.8 (C‐8′), 27.8 (C‐9′), 27.2 (C‐5′), 25.7 (C‐12′), 24.4 (C‐1′), 18.3 (C‐9), 17.7 (C‐15′), 16.1 (C‐13′), 16.0 ppm (C‐14′); ^19^F NMR (600 MHz, [D_4_]MeOD, 300 K): *δ*=109.3 ppm (ddd, *J*_F,H_=9.6, 8.6, 6.2 Hz, 1F, CF‐7); HRMS (ESI): *m/z* calcd for C_25_H_32_FNO: 382.2541 [*M*+H]^+^; found: 382.2549.

**6‐Fluoroaurachin D (1 c)**: ^1^H NMR (600 MHz, [D_4_]MeOD, 300 K): *δ*=7.84 (dd, *J*_H,H_=9.4, 2.9 Hz, 1H; CH‐5), 7.56 (dd, *J*_H,H_=9.1, *J*_H,F_=4.5 Hz, 1H, CH‐8), 7.45 (ddd, *J*_H,H_=9.1, 2.9, *J*_H,F_=8.1 Hz, 1H, CH‐7), 5.09 (dt, *J*_H,H_=6.9, 1.3 Hz, 1H, CH‐2′), 5.05 (dt, *J*_H,H_=6.9, 1.3 Hz, 1H, CH‐6′), 4.99 (ddt, *J*_H,H_=7.0, 1.5, 1.4 Hz, 1H, CH‐10′), 3.40 (d, *J*_H,H_=6.9 Hz, 2H, CH_2_‐1′), 2.47 (s, 3H, CH_3_‐9), 2.09 (m, 2H, CH_2_‐5′), 2.02 (m, 2H, CH_2_‐4′), 1.94 (m, 2H, CH_2_‐9′), 1.88 (m, 2H, CH_2_‐8′), 1.81 (d, *J*_H,H_=1.3 Hz, 3H, CH_3_‐13′), 1.61 (d, *J*_H,H_=1.4 Hz, 3H, CH_3_‐12′), 1.55 (d, *J*_H,H_=1.3 Hz, 3H, CH_3_‐14′), 1.51 ppm (d, *J*_H,H_=1.5 Hz, 3H, CH_3_‐15′); ^13^C NMR (150 MHz, [D_4_]MeOD, 300 K): *δ*=177.7 (C‐4), 160.5 (d, *J*_C,F_=241.9 Hz, C‐6), 150.0 (C‐2), 137.2 (C‐8a), 136.1 (C‐3′), 135.9 (C‐7′), 132.1 (C‐11′), 125.4 (C‐6′), 125.3 (C‐10′), 126.2 (C‐4a), 123.6 (C‐2′), 121.6 (C‐8), 121.3 (C‐7), 120.8 (C‐3), 110.1 (C‐5), 40.8 (C‐4′), 40.7 (C‐8′), 27.8 (C‐9′), 27.3 (C‐5′), 25.8 (C‐12′), 24.8 (C‐1′), 18.3 (C‐9), 17.6 (C‐15′), 16.3 (C‐13′), 16.1 ppm (C‐14′); ^19^F NMR (600 MHz, [D_4_]MeOD, 300 K): *δ*=119.2 ppm (ddd, *J*_F,H_=9.4, 8.1, 4.5 Hz, 1F, CF‐6); HRMS (ESI): *m/z* calcd for C_25_H_32_FNO: 382.2541 [*M*+H]^+^; found: 382.2541.

**Antibacterial activity screening**: An agar diffusion assay was performed with the purified compounds **1** and **1 a**–**c** to test their antimicrobial activities against *Escherichia coli* SG458, *Pseudomonas aeruginosa* SG137, *Staphylococcus aureus* (MRSA) 134/94 and *Mycobacterium vaccae* IMET 10670. For this purpose, 50 μL compound test solution (500 μg mL^−1^ in DMSO) was pipetted into agar plates with pre‐punched 9 mm holes. Ciprofloxacin (5 μg L^−1^) was used as positive control.

## Conflict of interest

The authors declare no conflict of interest.

## Supporting information

As a service to our authors and readers, this journal provides supporting information supplied by the authors. Such materials are peer reviewed and may be re‐organized for online delivery, but are not copy‐edited or typeset. Technical support issues arising from supporting information (other than missing files) should be addressed to the authors.

Supplementary
