# Supplementary material for: Biosynthetic Plasticity Enables Production of Fluorinated Aurachins
Source: Chembiochem. 2020 May 5;21(16):2268–73. doi: 10.1002/cbic.202000166 (PMC7496329; doi:10.1002/cbic.202000166)

ChemBioChem

Supporting Information

Biosynthetic Plasticity Enables Production of Fluorinated Aurachins

Angela Sester, Katrin Stüer-Patowsky, Wolf Hiller, Florian Kloss, Stephan Lütz, and Markus Nett*

Table of Content

Figure S1. MS chromatograms of *S. erecta* crude extracts. SI-2
Figure S2. ^{19}F NMR spectra (600 MHz, CD_3OD) of 8- (**1a**), 7- (**1b**) and 6-fluoroaurachin D (**1c**) SI-3
Figure S3. HPLC-UV spectrum and ESI-MS spectrum of 8-fluoroaurachin D (**1a**) SI-4
Figure S4. ^{1}H NMR spectrum (600 MHz, CD_3OD) of 8-fluoroaurachin D (**1a**) SI-5
Figure S5. ^{1}H-decoupled ^{13}C NMR spectrum (150 MHz, CD_3OD) of 8-fluoroaurachin D (**1a**) SI-6
Figure S6. ^{1}H, ^{13}C HSQC spectrum (CD_3OD) of 8-fluoroaurachin D (**1a**) SI-7
Figure S7. ^{1}H, ^{13}C HMBC spectrum (CD_3OD) of 8-fluoroaurachin D (**1a**) SI-8
Figure S8. HPLC-UV spectrum and ESI-MS spectrum of 7-fluoroaurachin D (**1b**) SI-9
Figure S9. ^{1}H NMR spectrum (700 MHz, CD_3OD) of 7-fluoroaurachin D (**1b**) SI-10
Figure S10. ^{1}H-decoupled ^{13}C NMR spectrum (175 MHz, CD_3OD) of 7-fluoroaurachin D (**1b**) SI-11
Figure S11. ^{1}H, ^{13}C HSQC spectrum (CD_3OD) of 7-fluoroaurachin D (**1b**) SI-12
Figure S12. ^{1}H, ^{13}C HMBC spectrum (CD_3OD) of 7-fluoroaurachin D (**1b**) SI-13
Figure S13. HPLC-UV spectrum and ESI-MS spectrum of 6-fluoroaurachin D (**1c**) SI-14
Figure S14. ^{1}H NMR spectrum (600 MHz, CD_3OD) of 6-fluoroaurachin D (**1c**) SI-15
Figure S15. ^{1}H-decoupled ^{13}C NMR spectrum (150 MHz, CD_3OD) of 6-fluoroaurachin D (**1c**) SI-16
Figure S16. ^{1}H, ^{13}C HSQC spectrum (CD_3OD) of 6-fluoroaurachin D (**1c**) SI-17
Figure S17. ^{1}H, ^{13}C HMBC spectrum (CD_3OD) of 6-fluoroaurachin D (**1c**) SI-18
Figure S18. ESI-MS spectrum of 6-chloroaurachin D (**1g**) SI-19

Figure S1. MS chromatograms of *S. erecta* crude extracts after feeding fluoroanthranilic acids. Base peak chromatogram with extracted ion chromatograms of compound **1** (grey) and compounds **1a** – **1d** (black).

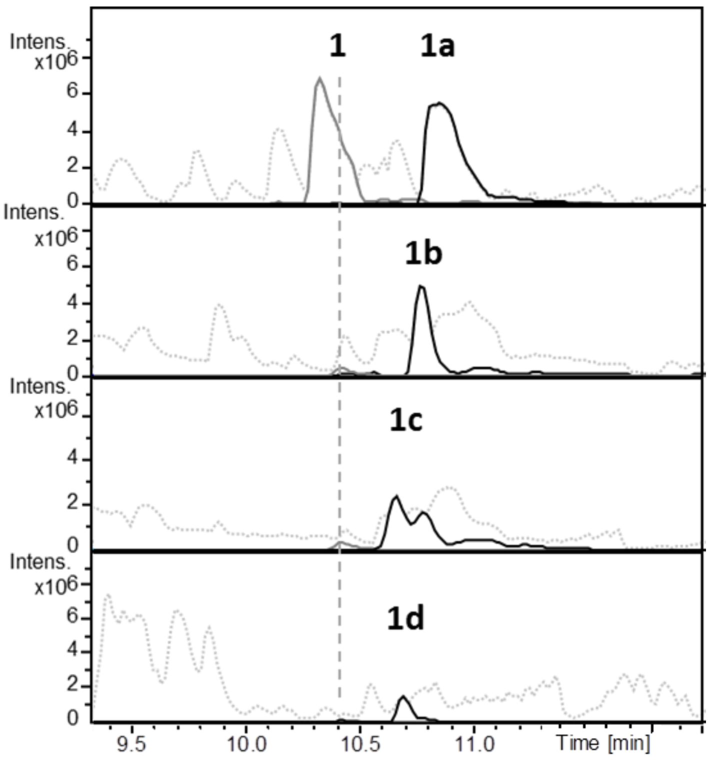

Table S1. Production yield of aurachin D (**1**) and **1**-derived metabolites (**1a-h**) after feeding of halogenated anthranilic acids.

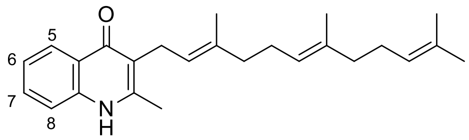

1a: 8 = F **1e:** 8 = Cl
1b: 7 = F **1f:** 7 = Cl
1c: 6 = F **1g:** 6 = Cl
1d: 5 = F **1h:** 5 = Cl

Supplement	Yield of **1** [µg L^{-1}]	Yield of halogenated derivative [µg L^{-1}]
-	8.3	-
anthranilic acid	83.3	-
3-fluoroanthranilic acid	40.5	53.3 (**1a**)
4-fluoroanthranilic acid	1.0	18.4 (**1b**)
5-fluoroanthranilic acid	1.3	43.8 (**1c**)
6-fluoroanthranilic acid	0.7	16.2 (**1d**)
3-chloroanthranilic acid	1.7	< 0.1 (**1e**)
4-chloroanthranilic acid	2.8	< 0.1 (**1f**)
5-chloroanthranilic acid	3.2	10.4 (**1g**)
6-chloroanthranilic acid	8.1	< 0.1 (**1h**)

Figure S2. ^{19}F NMR spectra (600 MHz, CD_3OD) of 8- (**1a**), 7- (**1b**) and 6-fluoroaurachin D (**1c**)

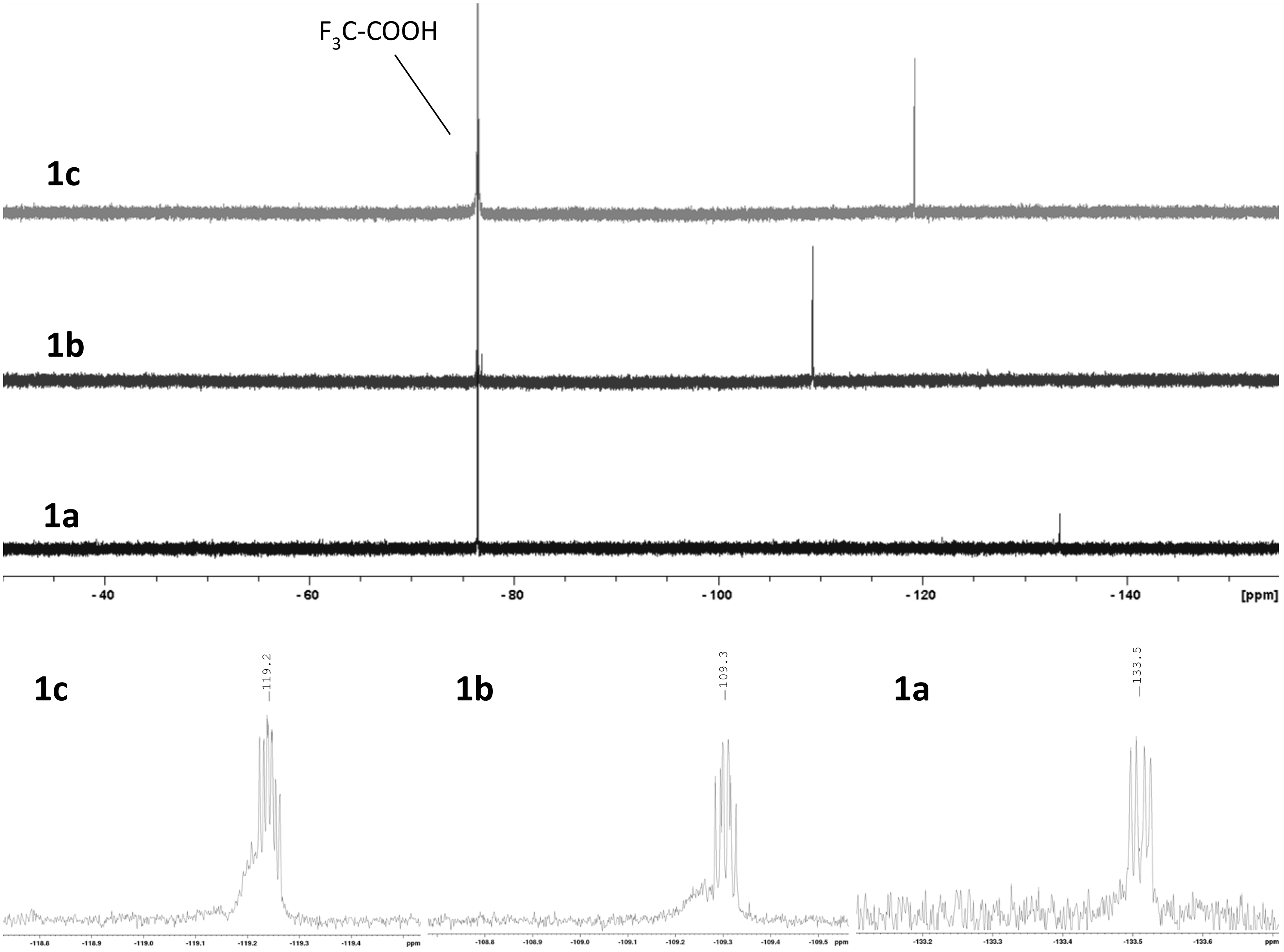

Figure S3. HPLC-UV spectrum and ESI-MS spectrum of 8-fluoroaurachin D (**1a**)

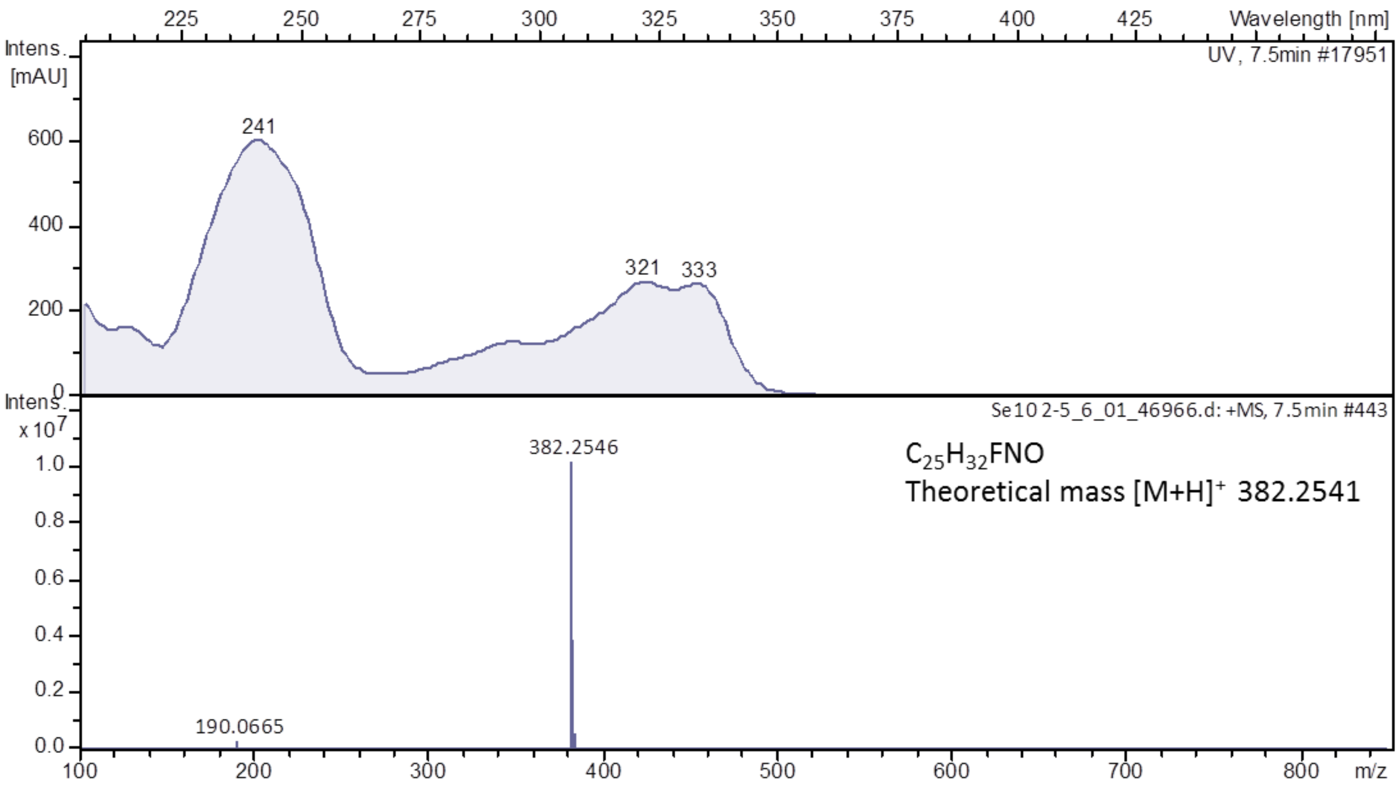

Figure S4. ^{1}H NMR spectrum (600 MHz, CD_3OD) of 8-fluoroaurachin D (**1a**)

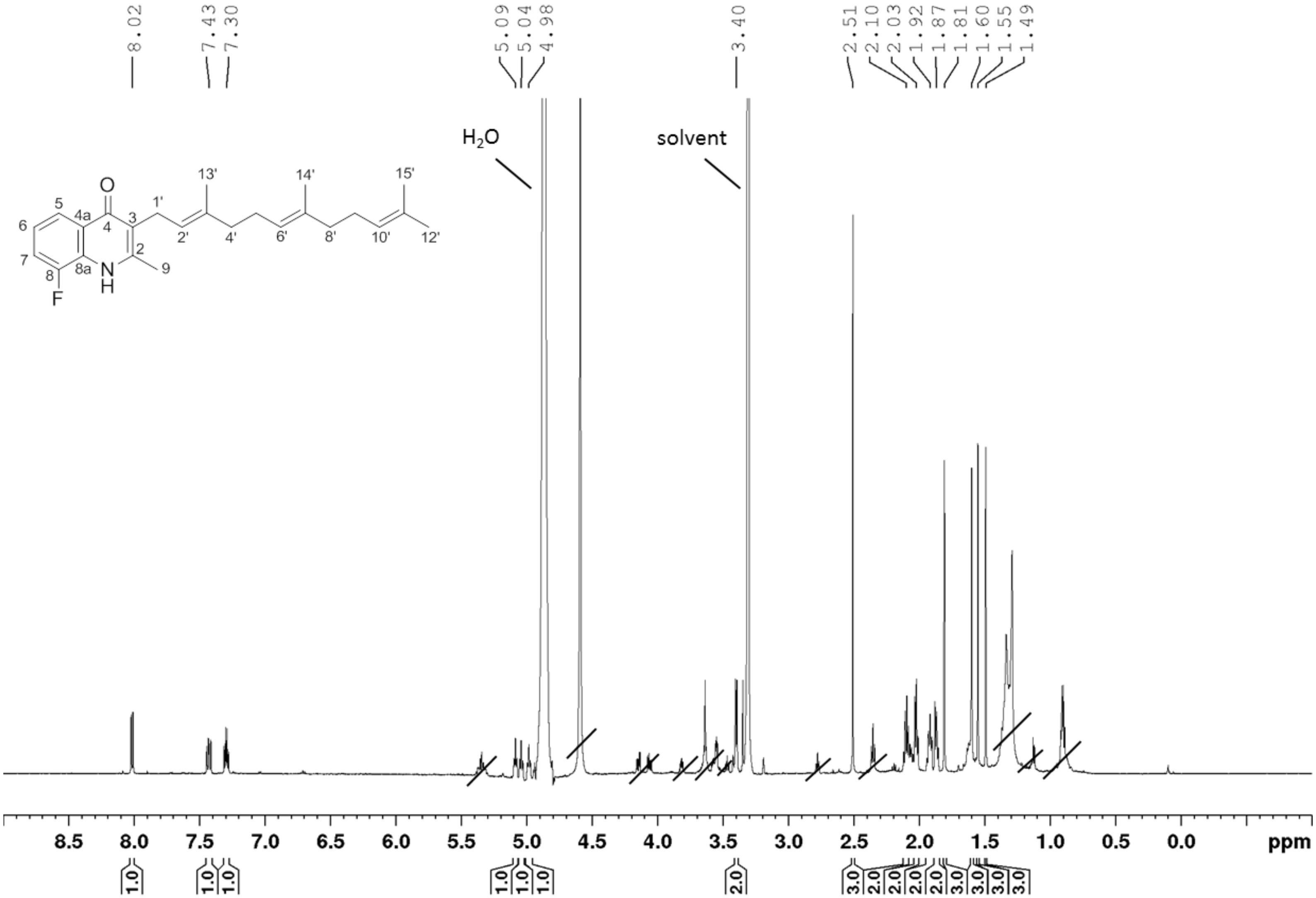

Figure S5. ^{1}H-decoupled ^{13}C NMR spectrum (150 MHz, CD_3OD) of 8-fluoroaurachin D (**1a**)

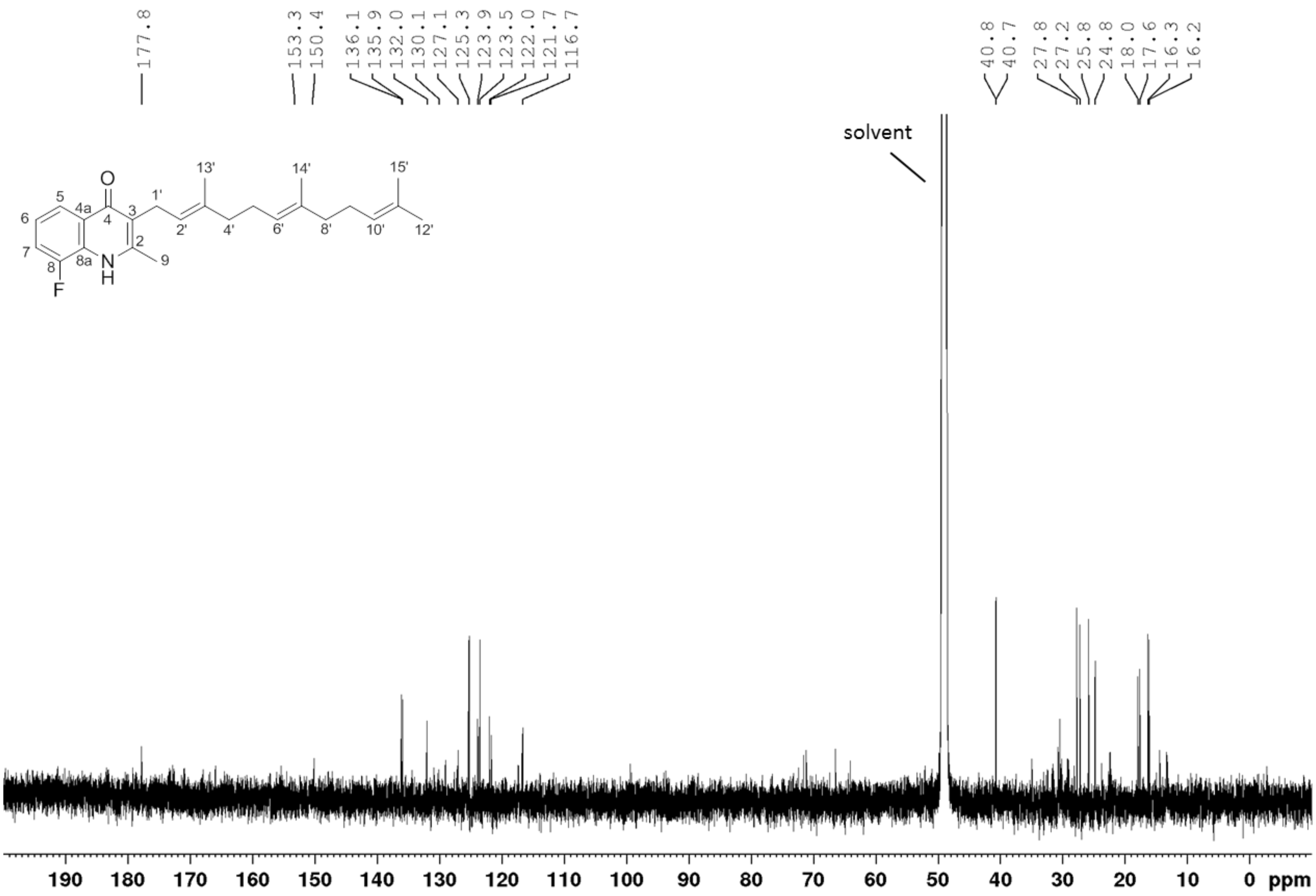

Figure S6. ^{1}H, ^{13}C HSQC spectrum (CD_3OD) of 8-fluoroaurachin D (**1a**)

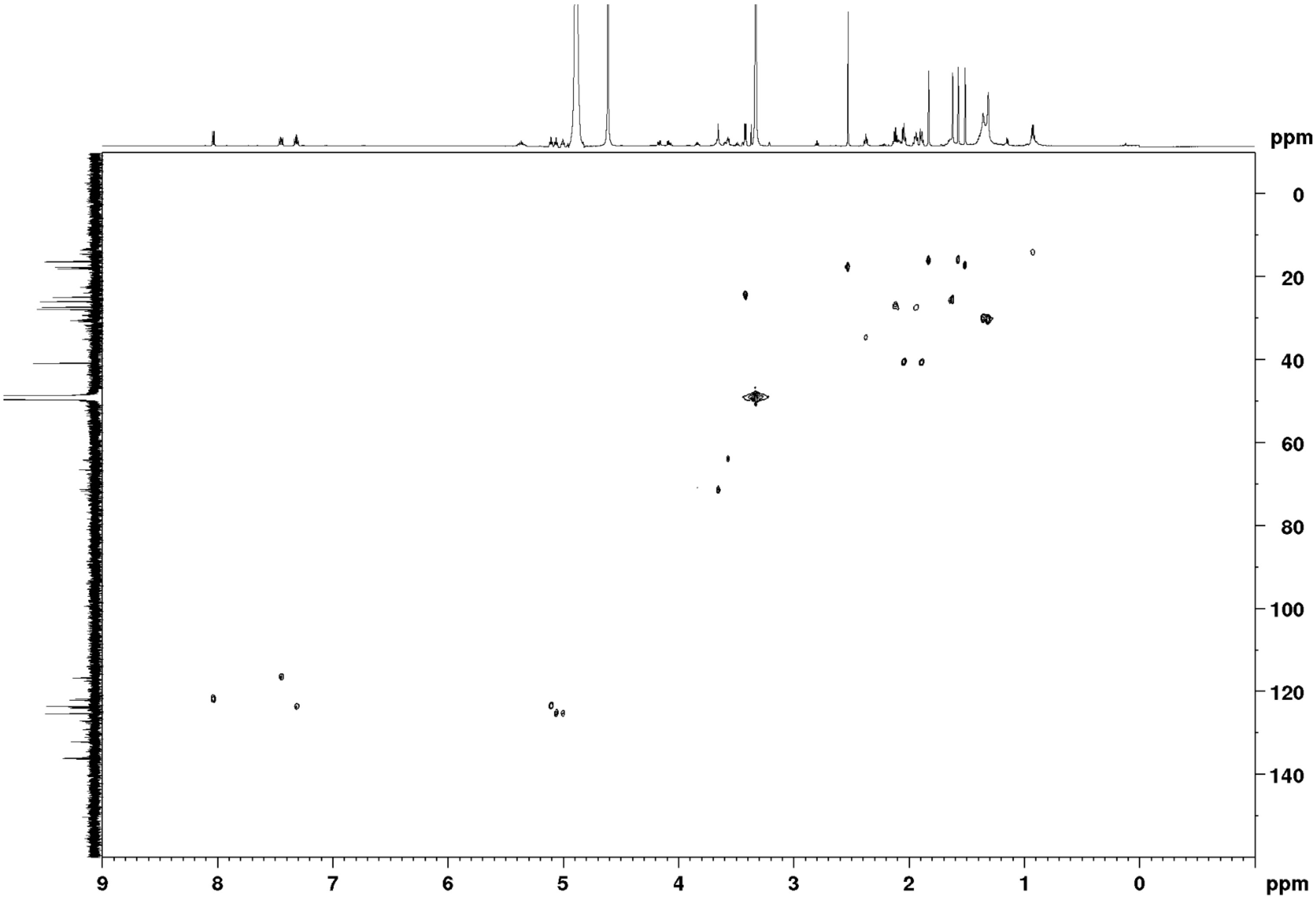

Figure S7. ^{1}H, ^{13}C HMBC spectrum (CD_3OD) of 8-fluoroaurachin D (**1a**)

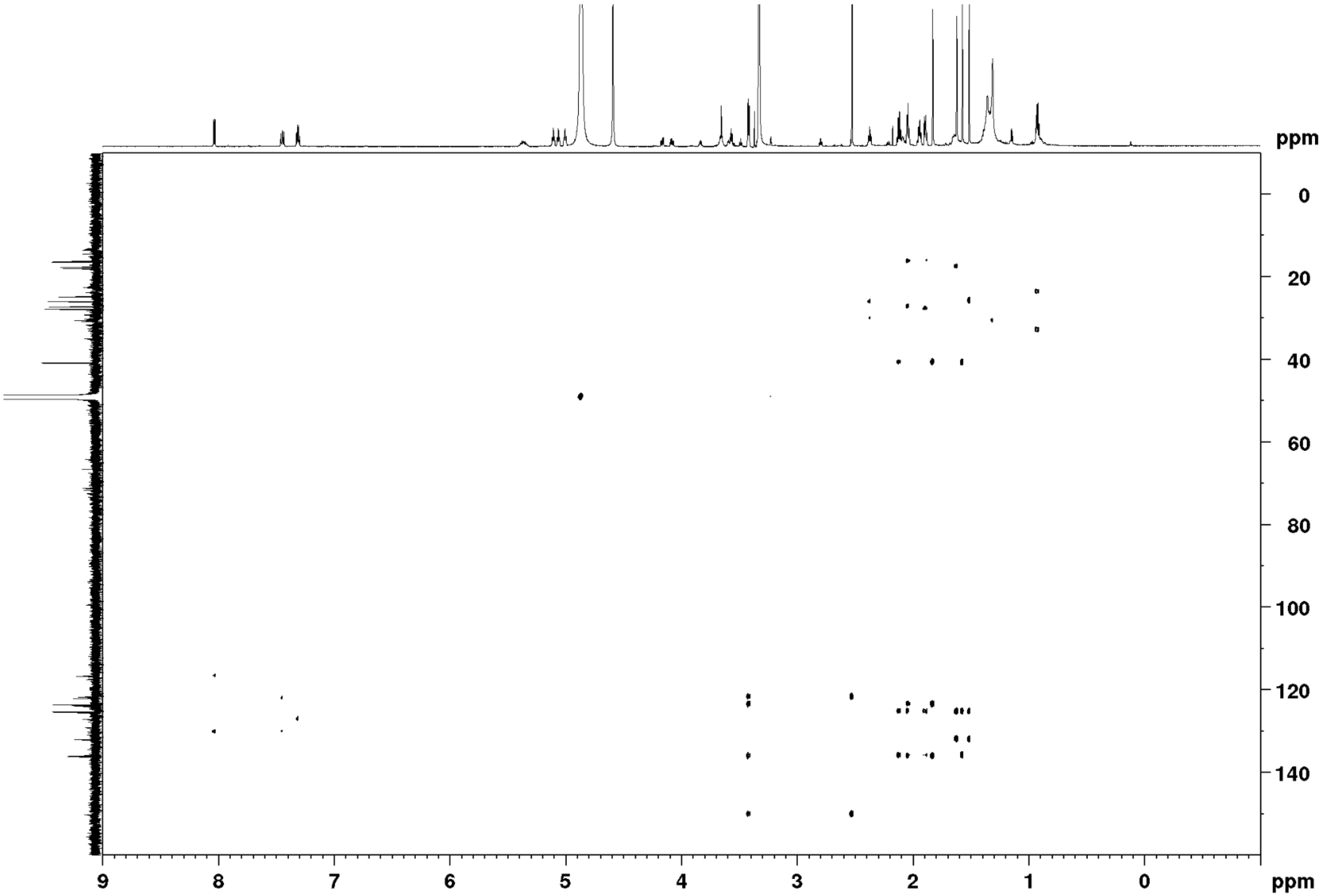

Figure S8. HPLC-UV spectrum and ESI-MS spectrum of 7-fluoroaurachin D (**1b**)

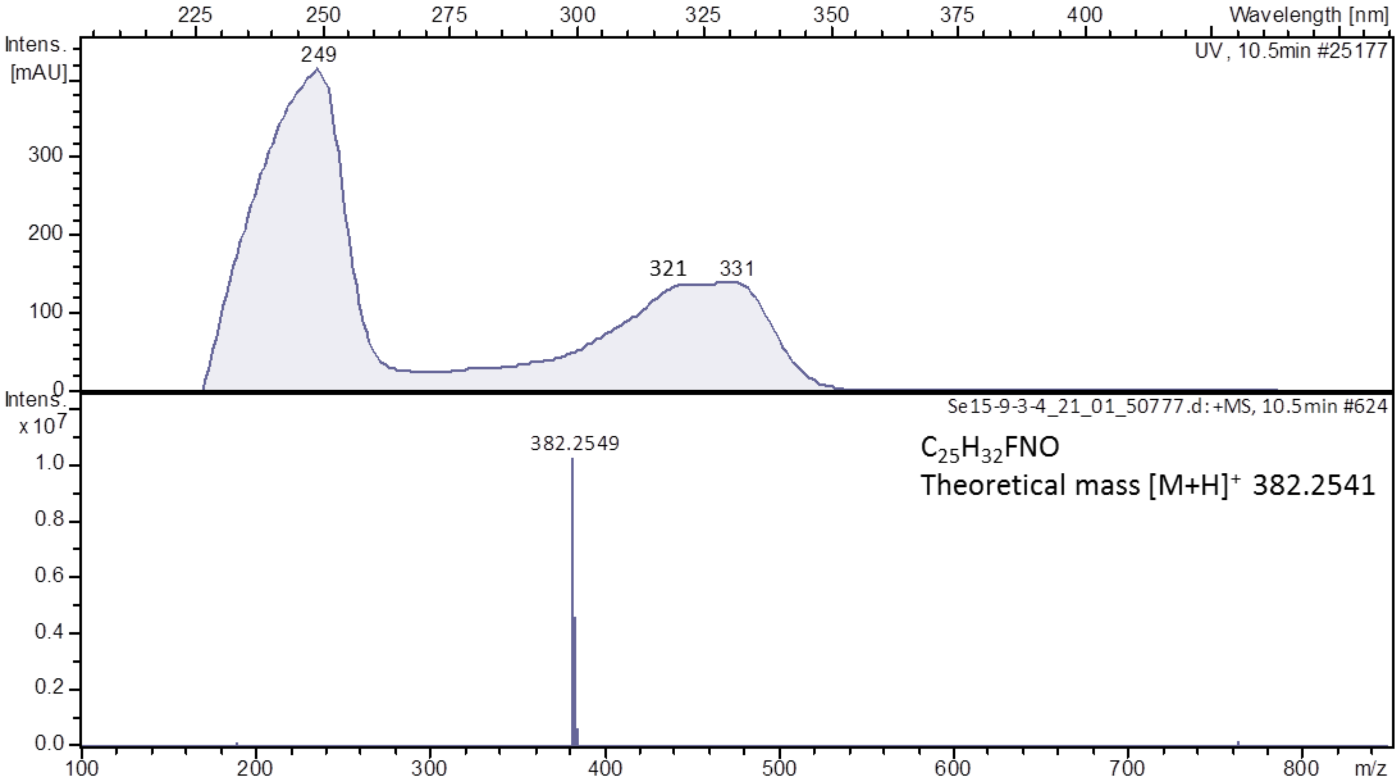

Figure S9. ^{1}H NMR spectrum (700 MHz, CD_3OD) of 7-fluoroaurachin D (**1b**)

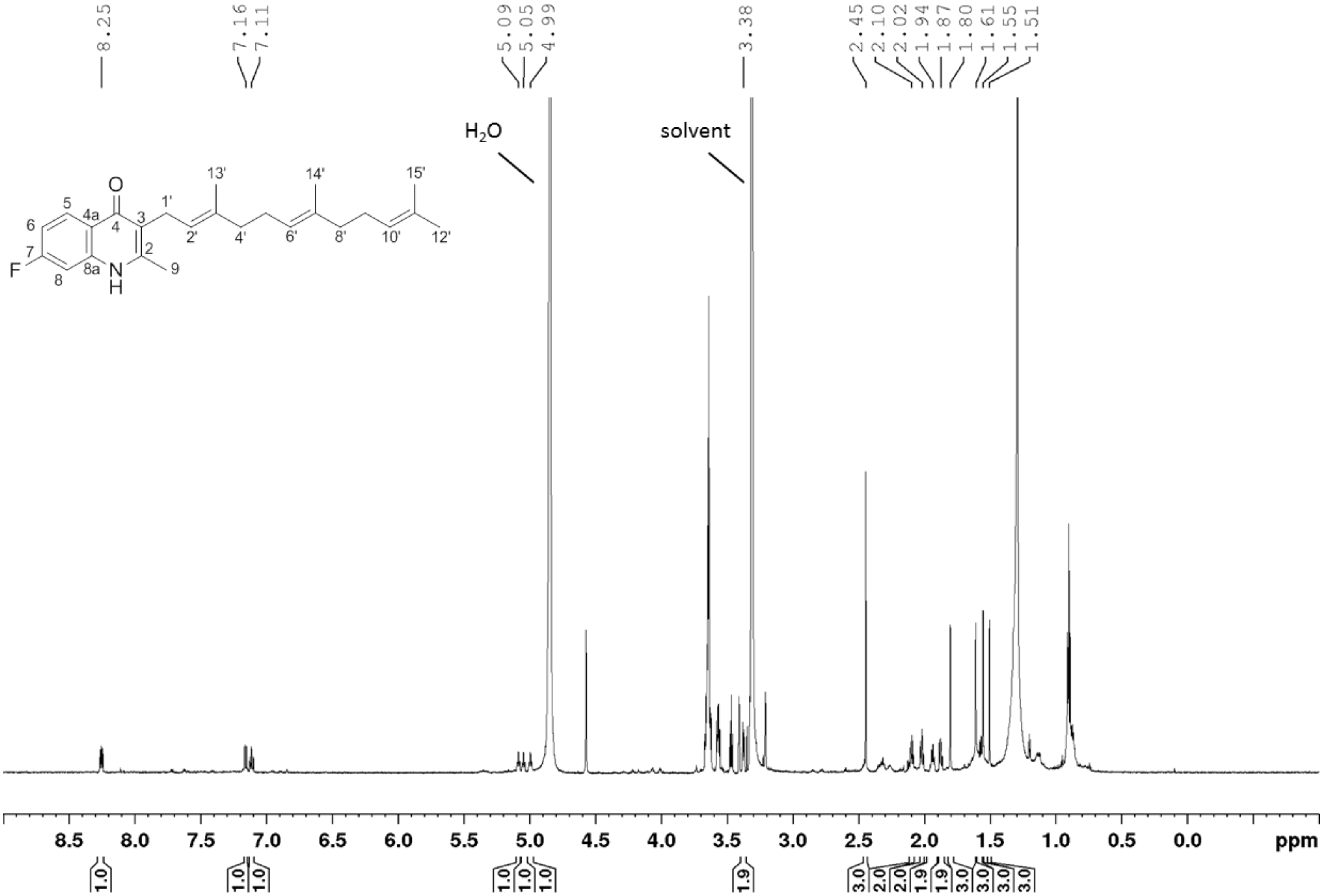

Figure S10. ^{1}H-decoupled ^{13}C NMR spectrum (175 MHz, CD_3OD) of 7-fluoroaurachin D (**1b**)

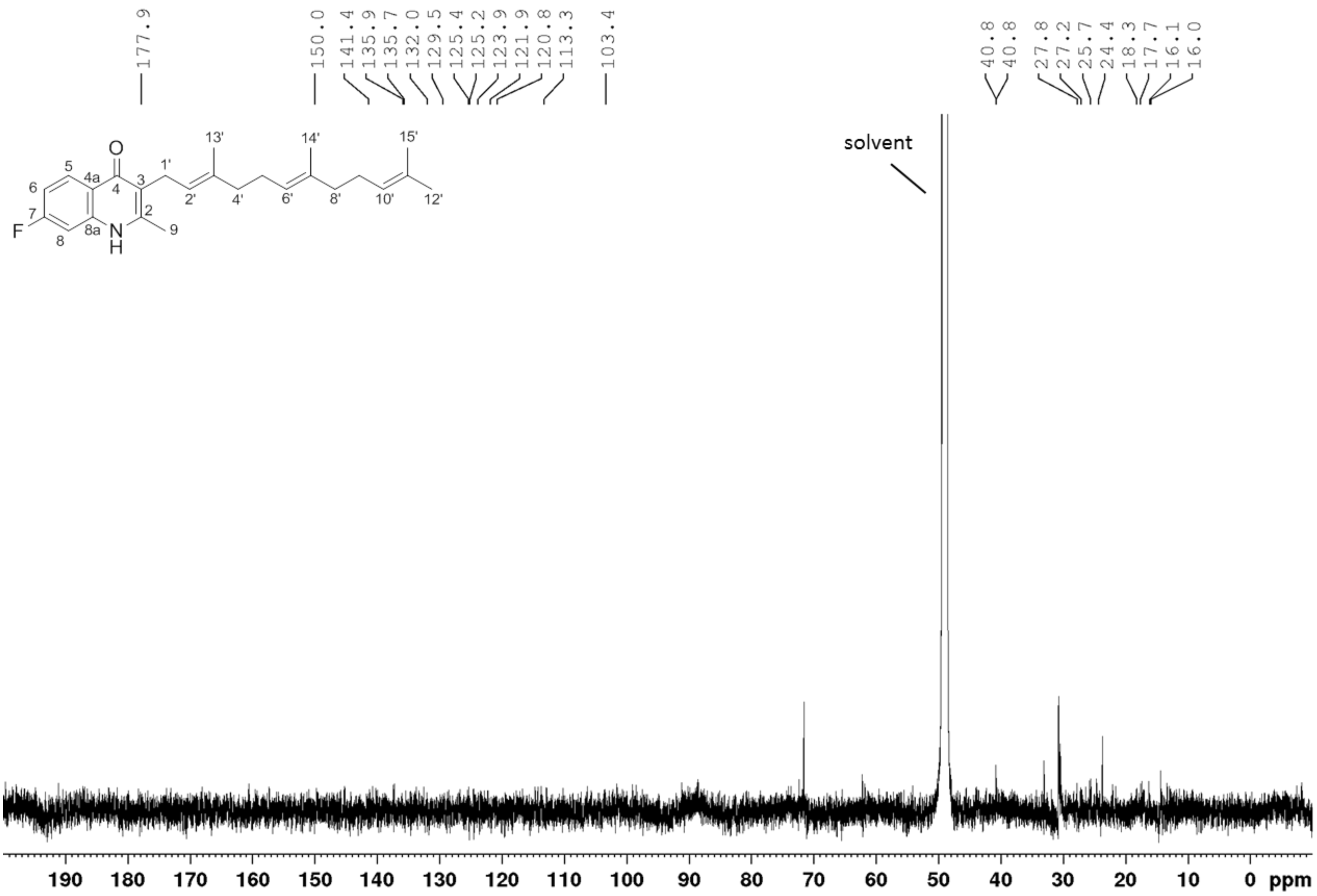

Figure S11. ^{1}H, ^{13}C HSQC spectrum (CD_3OD) of 7-fluoroaurachin D (**1b**)

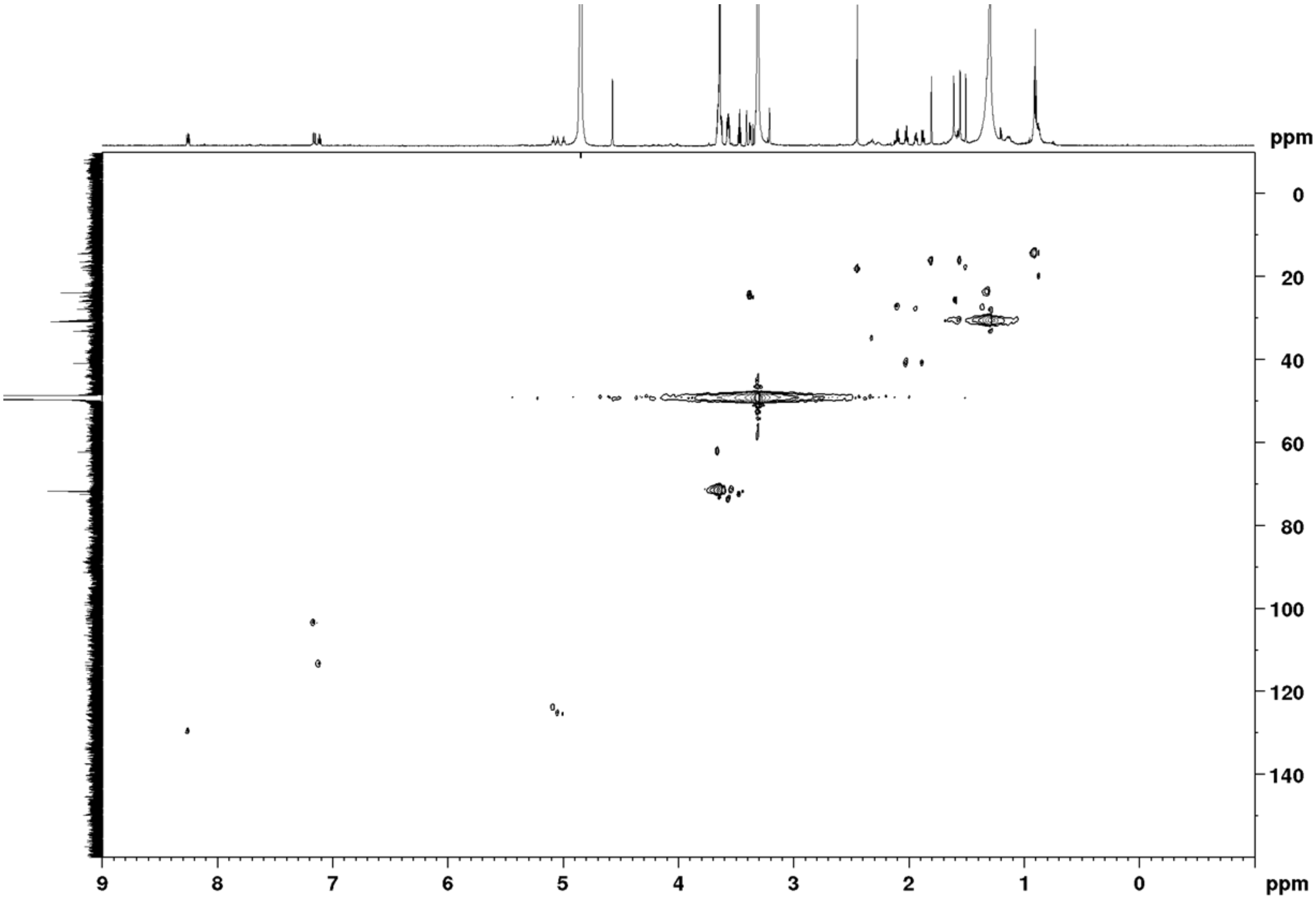

Figure S12. ^1H, ^{13}C HMBC spectrum (CD_3OD) of 7-fluoroaurachin D **(1b)**

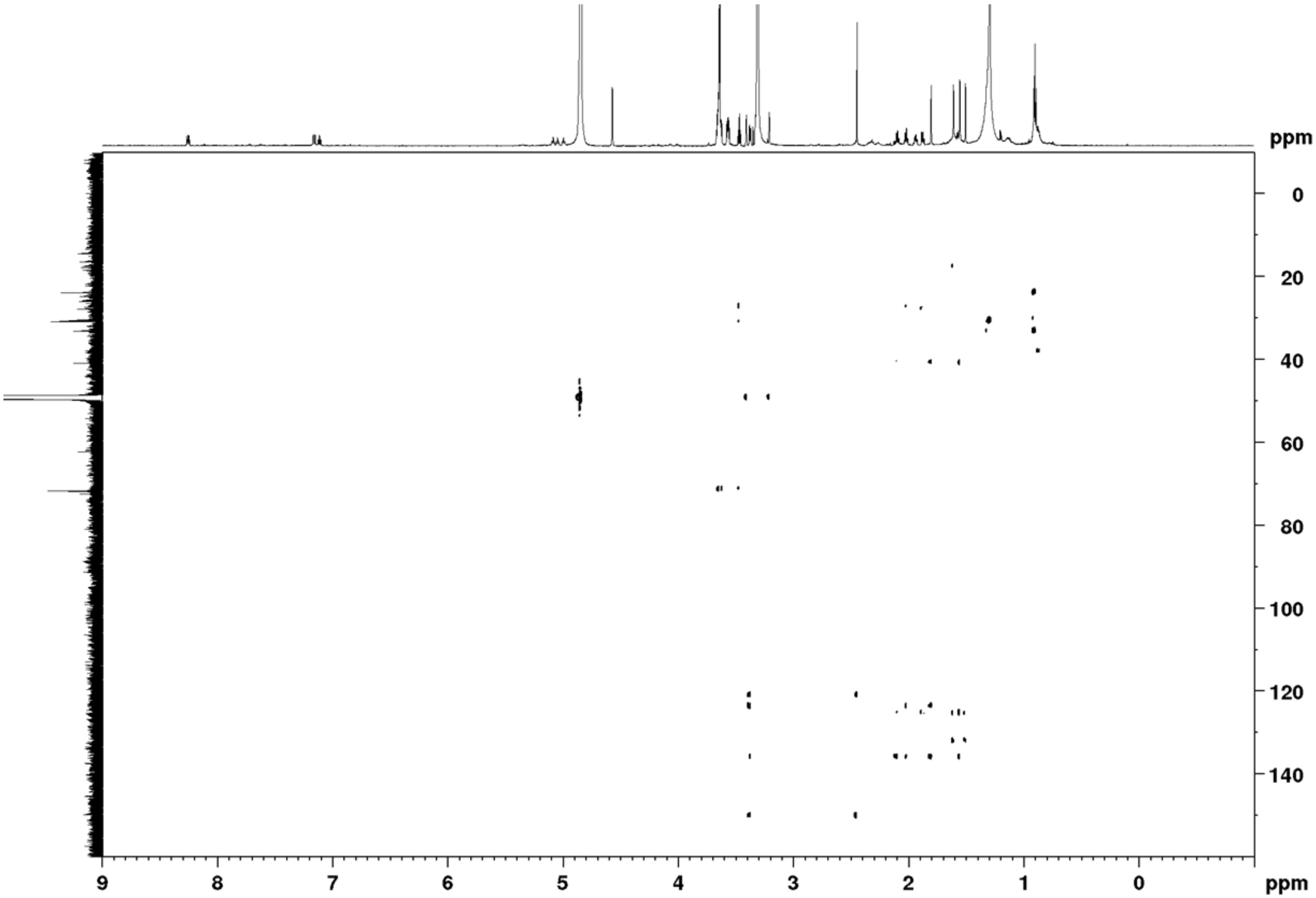

Figure S13. HPLC-UV spectrum and ESI-MS spectrum of 6-fluoroaurachin D (**1c**)

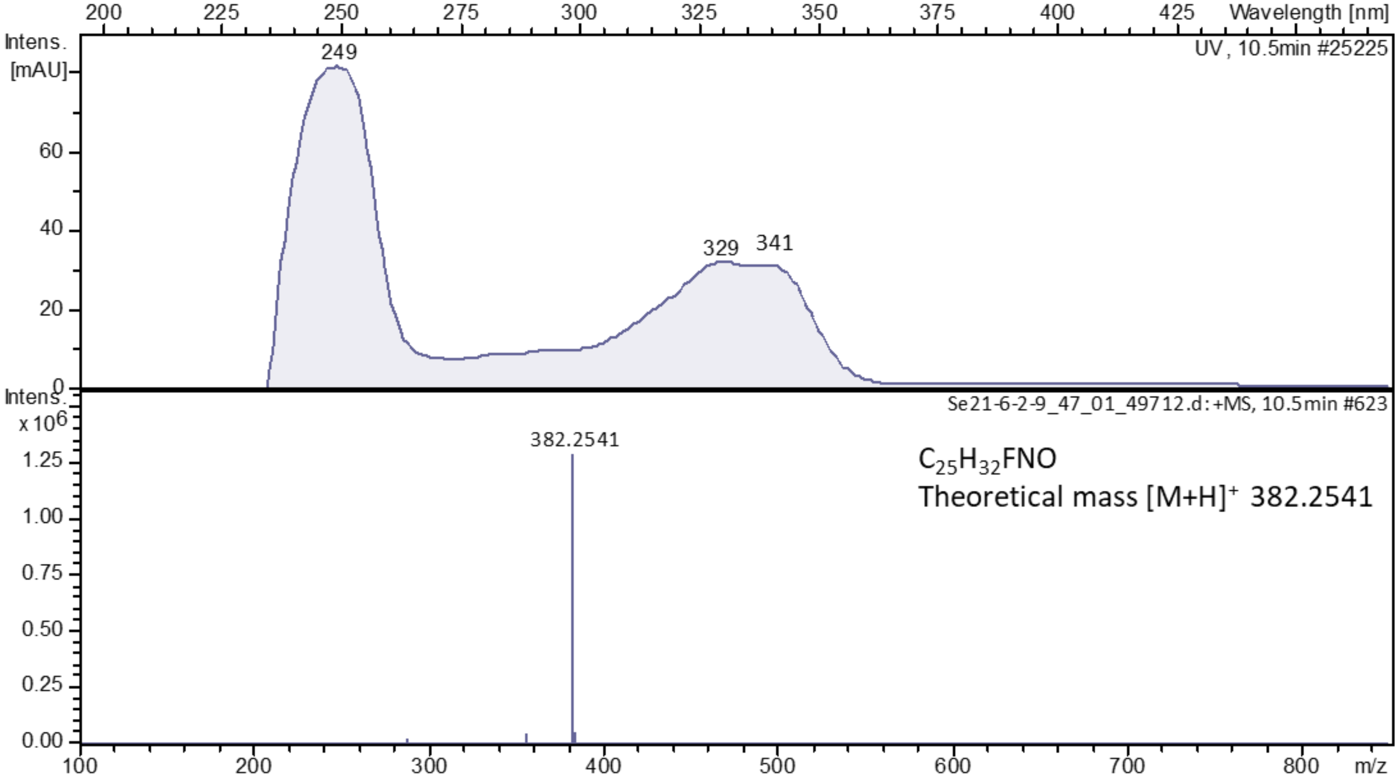

Figure S14. ^{1}H NMR spectrum (600 MHz, CD_3OD) of 6-fluoroaurachin D (**1c**)

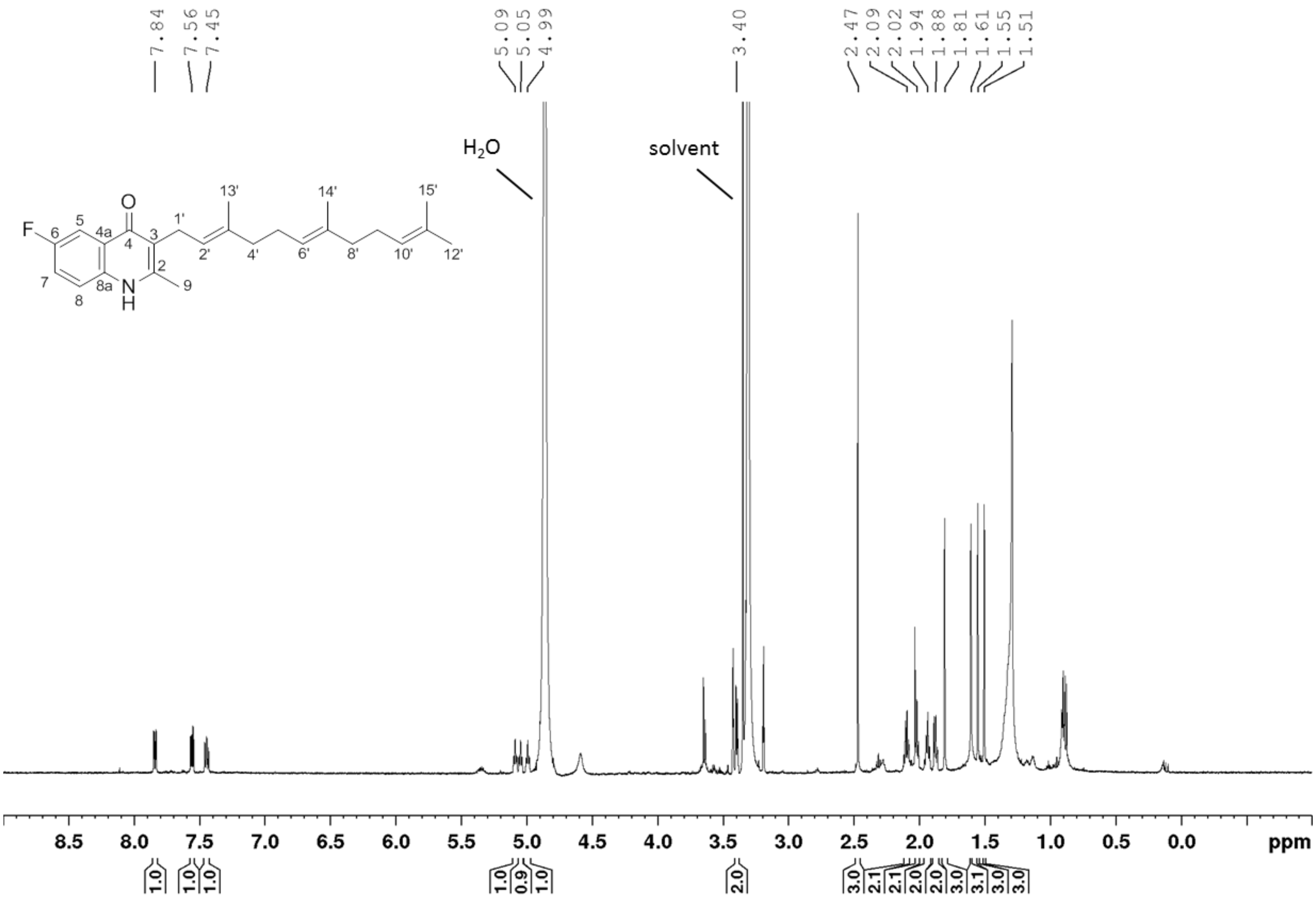

Figure S15. ^{1}H-decoupled ^{13}C NMR spectrum (150 MHz, CD_3OD) of 6-fluoroaurachin D (**1c**)

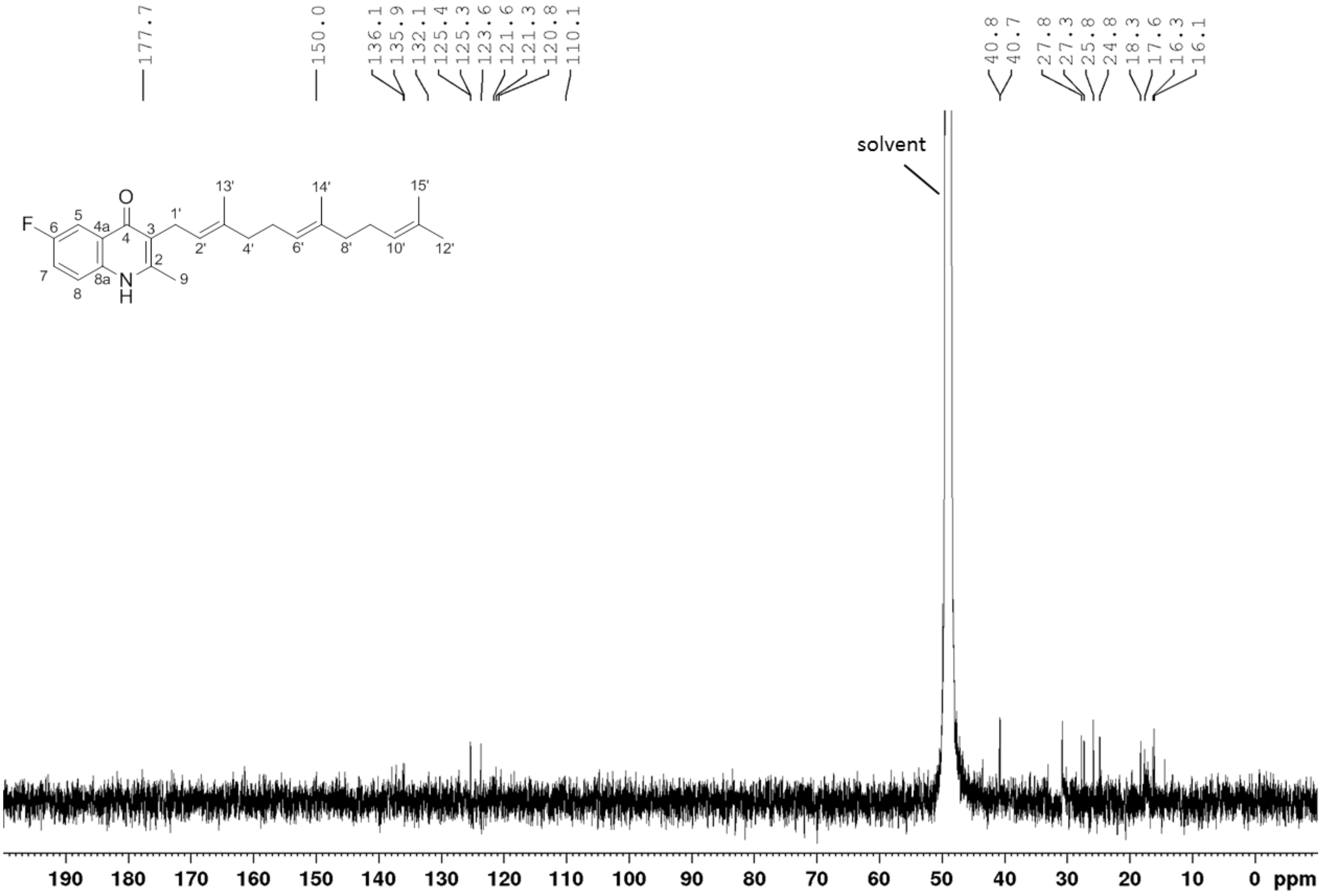

Figure S16. ^{1}H, ^{13}C HSQC spectrum (CD_3OD) of 6-fluoroaurachin D (**1c**)

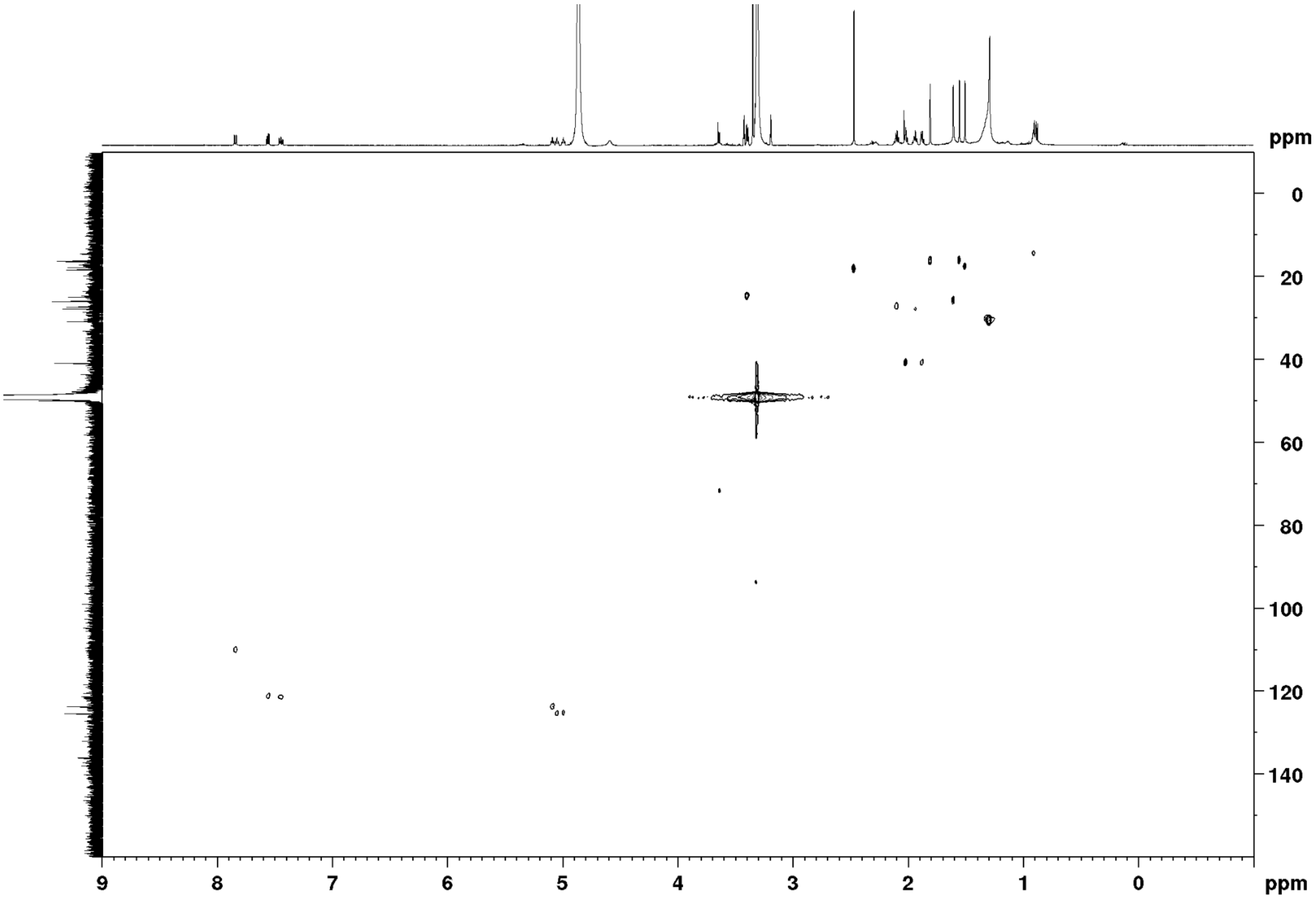

Figure S17. ^{1}H, ^{13}C HMBC spectrum (CD_3OD) of 6-fluoroaurachin D (**1c**)

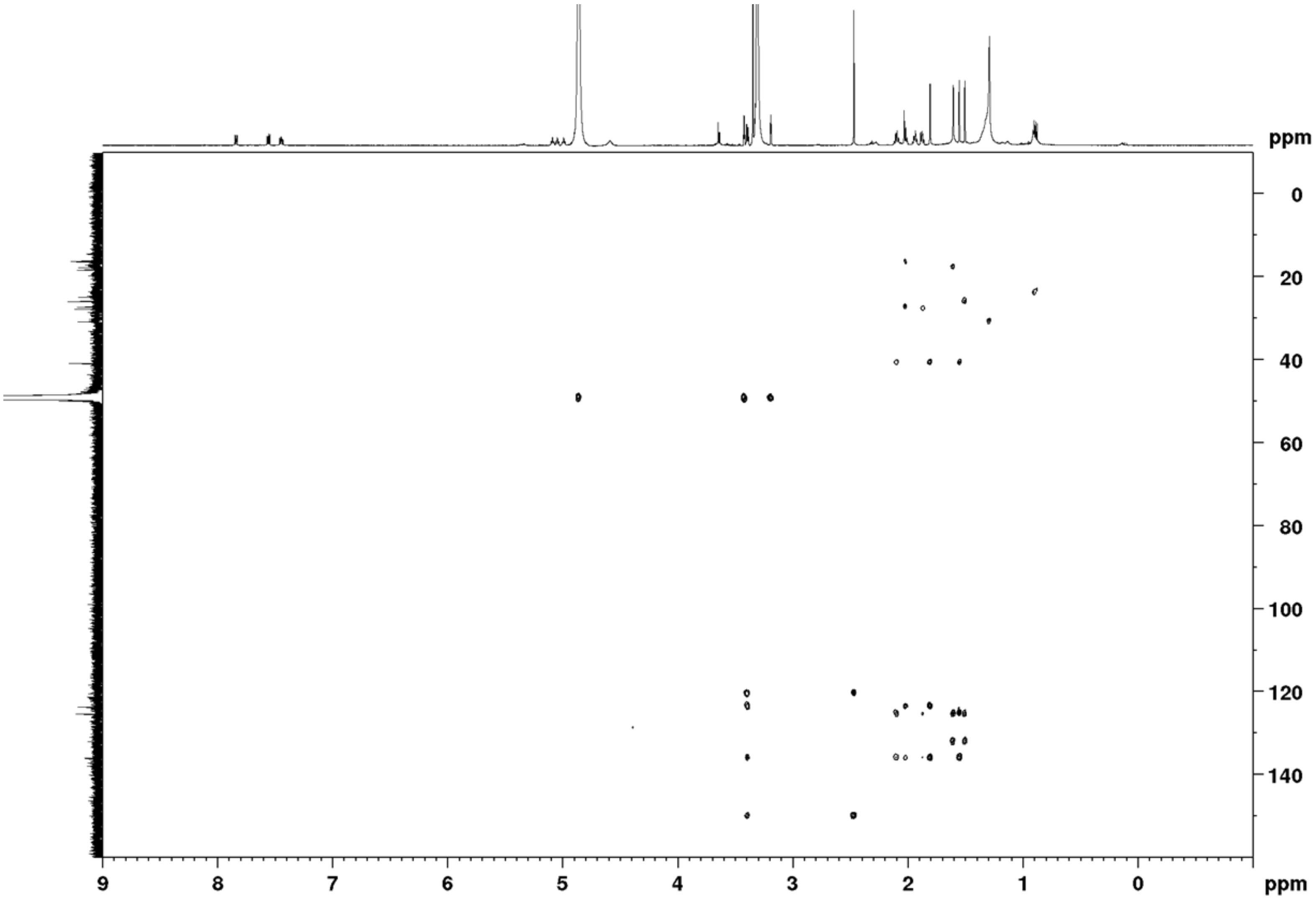

Figure S18. ESI-MS spectrum of 6-chloroaurachin D (**1g**)

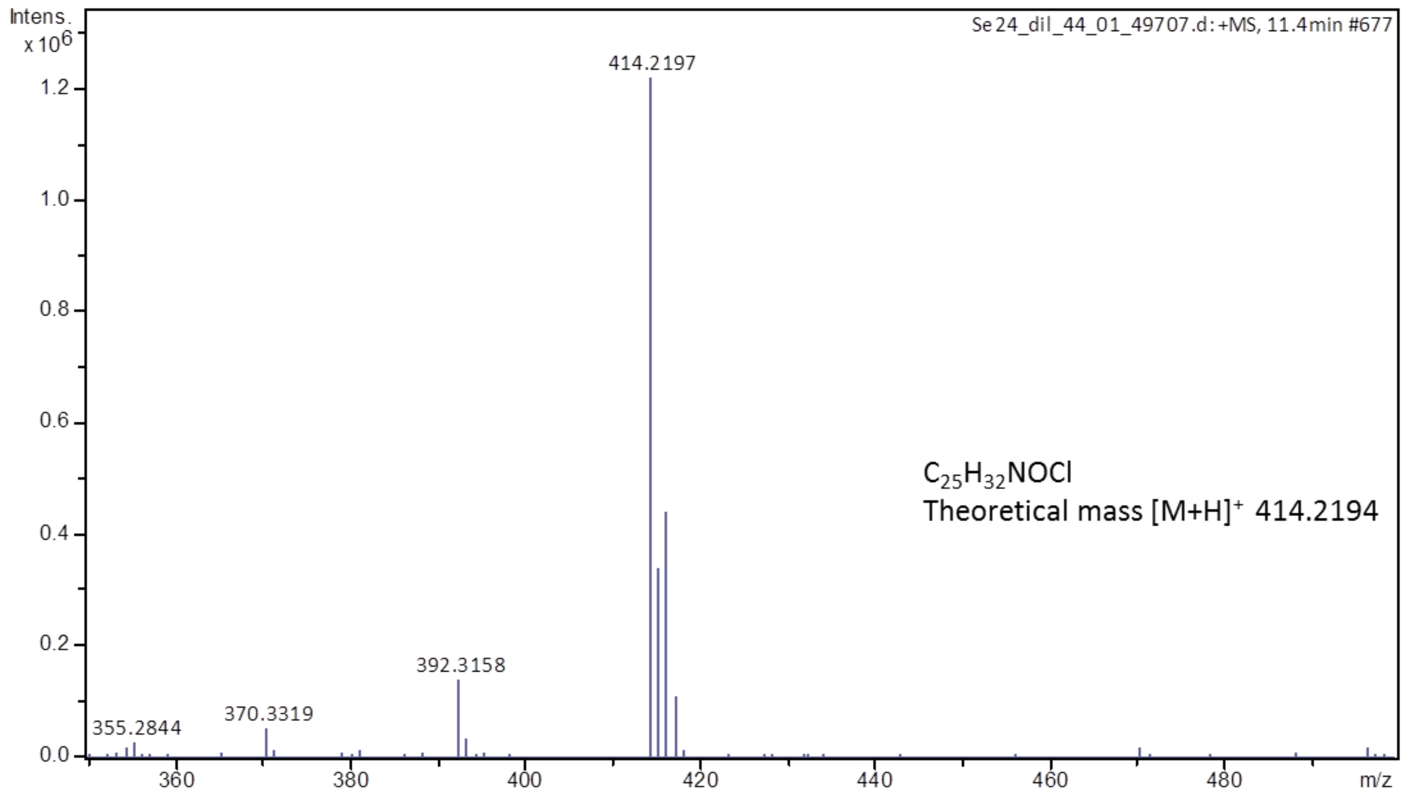

Supplement: Supplementary file 1 — Supplementary [file CBIC-21-2268-s001.pdf]
